# Syntheses and Reactivity of New Zwitterionic Imidazolium Trihydridoborate and Triphenylborate Species

**DOI:** 10.3390/molecules25143184

**Published:** 2020-07-13

**Authors:** Maura Pellei, Riccardo Vallesi, Luca Bagnarelli, H. V. Rasika Dias, Carlo Santini

**Affiliations:** 1 School of Science and Technology, Chemistry Division, University of Camerino, via S. Agostino 1, 62032 Camerino, Macerata, Italy; maura.pellei@unicam.it (M.P.); riccardo.vallesi@unicam.it (R.V.); luca.bagnarelli@unicam.it (L.B.); 2 Department of Chemistry and Biochemistry, The University of Texas at Arlington, Arlington, TX 76019-0065, USA

**Keywords:** *N*-heterocyclic carbenes, imidazole, spectroscopy, X-ray

## Abstract

In this study, four new *N*-(alkyl/aryl)imidazolium-borates were prepared, and their deprotonation reactions were investigated. Addition of BH_3_•THF to *N*-benzylimidazoles and *N*-mesitylimidazoles leads to imidazolium-trihydridoborate adducts. Ammonium tetraphenylborate reacts with benzyl- or mesityl-imidazoles with the loss of one of the phenyl groups yielding the corresponding imidazolium-triphenylborates. Their authenticity was confirmed by CHN analysis, ^1^H-NMR, ^13^C-NMR, ^11^B-NMR, FT-IR spectroscopy, and electrospray ionization mass spectrometry (ESI-MS). 3-Benzyl-imidazolium-1-yl)trihydridoborate, (HIm^Bn^)BH_3_, and (3-mesityl-imidazolium-1-yl)trihydridoborate, (HIm^Mes^)BH_3_, were also characterized by X-ray crystallography. The reactivity of these new compounds as carbene precursors in an effort to obtain borate-NHC complexes was investigated and a new carbene-borate adduct (which dimerizes) was obtained via a microwave-assisted procedure.

## 1. Introduction

*N*-heterocyclic carbenes (NHCs) [1,2,3] are an extremely useful and versatile class of ligands [4,5,6,7,8,9,10] with donor properties similar to phosphanes [11,12,13,14]. By tuning the steric and electronic properties around the carbene center, several carbenes featuring various σ-donating and π-accepting properties have been developed to date [15,16,17]. Their chemical versatility not only implies a wide variety of structural diversity and coordination modes, but also a capability to form stable complexes with a large number of transition metals with different oxidation states [6,7,18,19,20] and labile ligands [21,22,23,24,25,26]. Metal-NHCs complexes gained considerable interest in recent years because of their application in material chemistry [27], in catalysis [19,28,29,30,31,32,33,34,35,36,37], in carbene transfer reactions [38,39], and in medicinal inorganic chemistry [40,41,42,43,44,45,46,47,48,49].

**Scheme 1:**
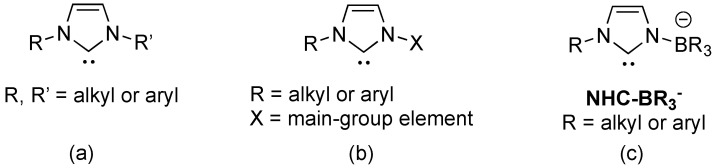
Structure of carbenes based on the imidazol-2-ylidene moieties: (**a**) with alkyl and aryl substituents; (**b**) with a main-group element substituent; (**c**) with a borate moiety substituent.

In the last thirty years, several carbenes based on the imidazol-2-ylidene (Scheme 1a) as well as the imidazolin-2-ylidene [50] and the chain-like carbene compound have been reported [51]. Such NHCs compounds have in common the presence of only organic substituents attached to the nitrogen atoms, whereas carbenes with other main-group elements as substituents (Scheme 1b) are scarce [52,53,54,55,56]. Substitution of one of the groups attached to nitrogen by a borane would result in the generation of carbene-borate anions NHC-BR_3_^−^ (Scheme 1c), as anionic analogs of the neutral imidazol-2-ylidenes. To the best of our knowledge, only few examples of monoanionic carbenes such as the on in Scheme 1c have been published as yet [53,57,58,59,60,61,62,63].

**Scheme 2:**
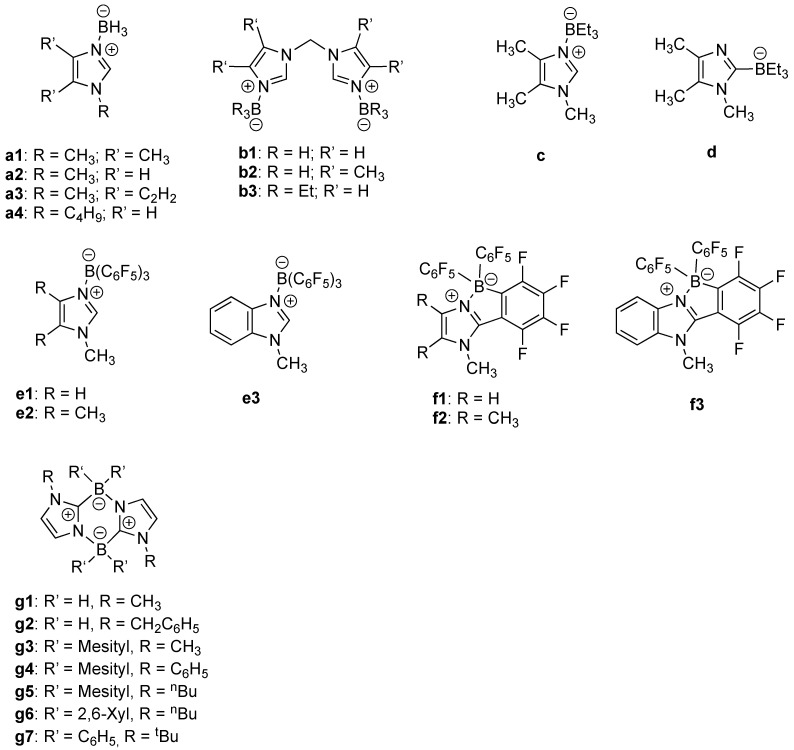
Structure of: (**a**) imidazolium trihydridoborate species; (**b**) bis(imidazolium)borate species; (**c**) imidazolium triethylborate species; (**d**) 2-substituted imidazolylborate species; (**e**) imidazolium and benzimidazolium triarylborate species; (**f**) ring-closed imidazolium and benzimidazolium triarylborate species; (**g**) imidazaboles.

In 1998 and 2002, Siebert and co-workers reported that deprotonation of imidazole-borane complexes or imidazolium-borate species (Scheme 2a) with BuLi leads to the formation of the carbene-borate anions NHC-BH_3_^−^ [59,64]. These kinds of nucleophilic carbenes allowed the formation of neutral manganese complexes and anionic iron compounds by reactions with BrMn(CO)_5_ and Fe(CO)_5_, respectively [59]. The analogous reaction with [(C_7_H_11_)Fe(CO)_2_Br], Cp_2_TiCl, VCl_3_, and ScCl_3_ yielded the corresponding metal complexes [64]. Bis(imidazolyl) compounds with BH_3_ or BEt_3_ (Scheme 2b) and their behavior towards treatment with butyllithium to give dianionic chelating dicarbene-diborate ligands have also been reported [53]. Among them, the dianionic bis(imidazol-2-ylidene) species obtained from b2 (Scheme 2) reacted with Cp_2_TiCl_2_ and Cp_2_ZrCl_2_ allowing the formation of the corresponding carbene-borate complexes [53]. Isomerization to the 2-borate imidazole forms by 1,2-BR_3_ migration [65], intramolecular addition/elimination or dimerization reactions may or may not occur on deprotonation [57,58,59,60,61,66]. For example, deprotonation of the triethylborane adduct (Scheme 2c) produced the isomerized *N*-heterocyclic carbene-borate species (Scheme 2d) [59]. Attempts to synthesize the carbene-borate anions by deprotonation of the parent imidazole (Scheme 2(e1,e2)) and benzimidazole (Scheme 2(e3)) adducts, have invariably resulted in the formation of isomers (Scheme 2f) [57,67] by ring-closure due to a rapid intramolecular nucleophilic aromatic substitution. On the other hand, Contreras et al. [60,66] reported the imidazaboles (Scheme 2(g1,g2)), by elimination of H_2_ from the (*N*-alkylimidazolium)borate species with iodine at 270 °C. Okada et al. [61] reported the synthesis of analogous imidazaboles (Scheme 2(g3,g4)), from reaction of the parent (*N*-alkylimidazolium)borates with organolithium reagents. Recently, Chiu and coworkers [65] reported that dimerization of 2-borylimidazoles through B−N coordination yielded the head-to-tail dimers g5 and g6 (Scheme 2). Compound g7 is the only isolable product of the reaction of [Ph_2_B(Im^t^Bu)_2_Br] and [Ca{N(SiMe_3_)_2_}_2_(THF)_2_] [68].

Functionalized imidazole-based NHCs have attracted special interest because they can be utilized to tune the environment and properties at the coordinated metal [4,69]. Whereas there are many studies describing the coordination of chelate and pincer *N*-heterocyclic carbene ligands, the use of anionic NHC-borates is still scarce [52,63]. Recently, significant research efforts have been devoted to the development of ionic liquids based on (*N*-alkylimidazolium)borate as new potential hypergolic fuels owing to their excellent physiochemical properties including and unique hypergolic reactivity [70]. The first chelating tricarbene ligand with the topology of Trofimenko’s tris(pyrazolyl)borates [71,72], tris(3-methylimidazolin-2-ylidene-1-yl)borate, in which the carbene units are connected via a BH group, was introduced in 1995 by Fehlhammer and co-workers [54] together with its hexacarbene iron(III) and cobalt(III) complexes [73,74]. The synthesis of monoanionic chelating dicarbene bis(imidazol-2-ylidene-1-yl)borates and their use as ligands in various homoleptic and heteroleptic metal complexes has been described [75,76] and recently reviewed [15,52].

In the last years, we developed several classes of coinage metal NHCs complexes obtained from the chelating precursors [HB(RImH)_3_]Br_2_ (R = Benzyl, Mesityl and t-Butyl) [77], [H_2_B(HTz^Bn^)_2_]Br [78], H_2_C(HTz^R^)_2_, and H_2_C(HIm^R^)_2_ (HTz = 1,2,4-triazole; HIm = imidazole; R = (CH_2_)_3_SO_3_^−^ or (CH_2_)_2_COO^−^) [79]. Recently, we have focused the research work on the development of new group 11 metal-NHCs complexes obtained from the water-soluble precursors HIm^1R,3R^Cl (R = COOCH_3_, COOCH_2_CH_3_, or CON(CH_2_CH_3_)_2_) [80,81] or the zwitterionic water-soluble precursor NaHIm^1R,3R^ (R = (CH_2_)_3_SO_3_^−^) [82].

Despite the impressive chemistry based on parent poly(azolyl)borate, the analogous mono(azolyl)borate have received very little attention in recent years [83,84]. Recently, we prepared trihydro(pyrazolyl)borates such as Na[H_3_B(5-(CF_3_)pz)] and Na[H_3_B(3-(NO_2_)pz)] and related copper(I) and silver(I) phosphane complexes [85,86].

**Scheme 3:**
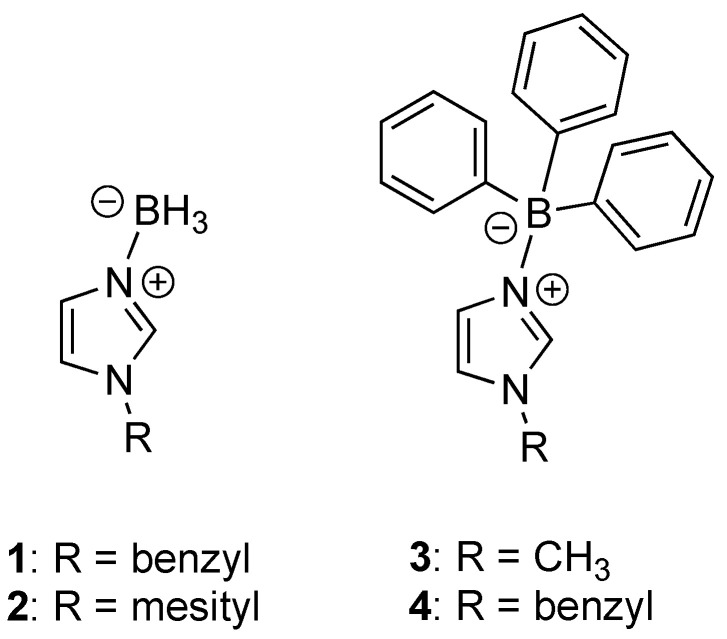
Chemical structures of (*N*-(alkyl/aryl)imidazolium)borates **1**–**4**.

Here, we present the synthesis of (*N*-(alkyl/aryl)imidazolium)borate-based systems (Scheme 3) and their reactivity as carbene precursors in the effort to obtain borate-NHCs silver(I) complexes.

## 2. Result and Discussion

### Synthesis and Characterization

**Scheme 4:**
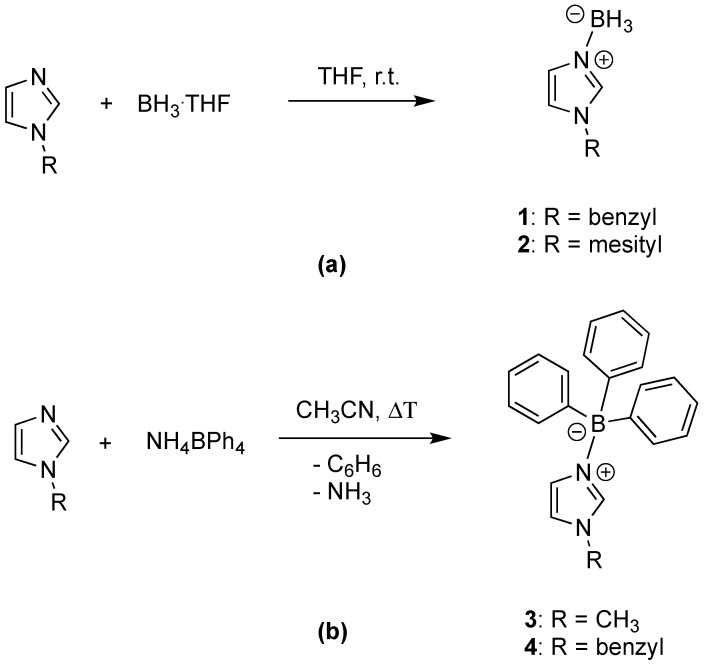
Synthesis of: (**a**) imidazolium trihydridoborates **1** and **2**; (**b**) imidazolium triphenyborates **3** and **4**.

The *N*-(alkyl/aril)imidazolium-borate adducts **1**–**4** were synthesized in one step by two different routes (Scheme 4).

The addition of one equivalent of BH_3_•THF to a solution of *N*-benzylimidazole or *N*-mesitylimidazole at room temperature yields the colorless imidazolium-borate adducts **1** or **2**, respectively, in nearly quantitative yields (Scheme 4a). By dissolving the crude ligands **1** and **2** in CHCl_3_ and CHCl_3_/THF solution, respectively, single crystals suitable for X-ray diffraction analysis were obtained.

Compounds **3** and **4** were prepared by addition of NH_4_BPh_4_ to an acetonitrile solution of methylimidazole or benzylimidazole under reflux conditions. It is known that under acidic conditions the tetraphenylborate anion has limited stability producing triphenylboranes [87], and when heated with alkylammonium salts can lose a phenyl ring to form a B–N bond with the ammonium compound [88]. This kind of displacement was observed in our studies: the loss of a phenyl ring and the formation of imidazolium-triphenylborate species occurred in good yields, volatile benzene and ammonia being also produced. Compound **3** was previously obtained as a crystalline byproduct of the reaction mixture of [ReO_2_(1-MeIm)_4_]^+^ complex and NaBPh_4_ in acidic conditions [89].

Derivatives **1** and **2** are white and brownish solids, respectively, both soluble in CH_3_OH, CHCl_3_, CH_2_Cl_2_, THF, DMSO, and acetone. Derivatives **3** and **4** are white solids, both soluble in THF, CH_2_Cl_2_, CHCl_3_, CH_3_CN, DMSO, and acetone.

The authenticity of compounds **1**–**4** was confirmed by CHN analysis, ^1^H-NMR, ^13^C-NMR, ^11^B-NMR, FT-IR spectroscopy, and electrospray ionization mass spectrometry (ESI-MS). Compounds **1** and **2** were also characterized by X-ray crystallography.

**Figure 1:**
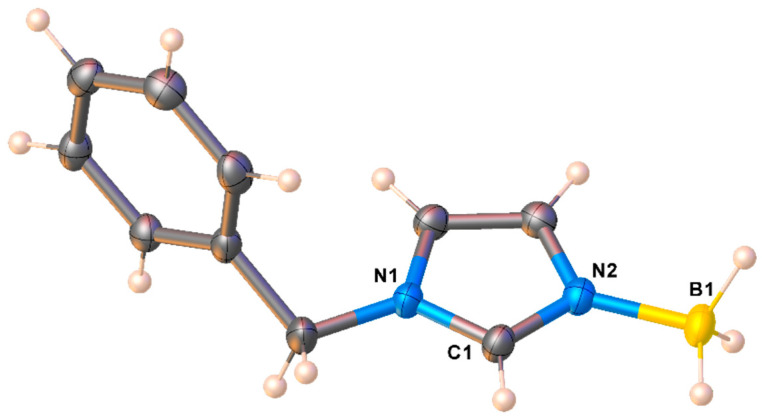
Molecular structure of (HIm^Bn^)BH_3_ (**1**). Selected bond distances (Å) and angles (**˚**): N1-C1 1.343(2), N2-C1 1.323(2), N2-B1 1.587(2), N1-C4 1.474(2), N1-C1-N2 110.29(14), C1-N2-B1 126.58(14).

The (HIm^Bn^)BH_3_ (**1**) crystallizes in the Orthorhombic P2_1_2_1_2_1_ space group. The molecular structure is illustrated in Figure 1. It is monomeric in the solid state and C1-N1 distance is slightly longer than the C1-N2 distance.

**Figure 2:**
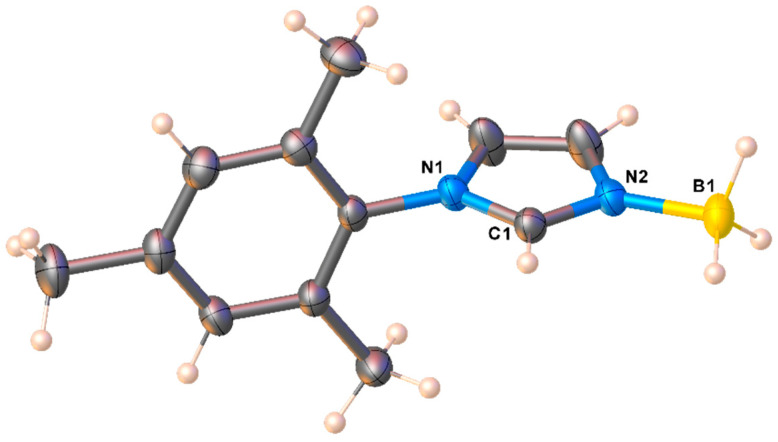
Molecular structure of (HIm^Mes^)BH_3_ (2). There are two chemical similar but crystallographically different molecules of (HIm^Mes^)BH_3_ in the asymmetric unit but only one is shown here. Selected bond distances (Å) and angles (˚): N1-C1 1.3442(14), N2-C1 1.3207(14), N2-B1 1.5836(16), N1-C4 1.4465(14), N1-C1-N2 110.36(10), C1-N2-B1 127.91(10).

The molecular structure of (HIm^Mes^)BH_3_ (**2**) is shown in Figure 2. It crystallizes in the Monoclinic P2_1_/*n* space group with two chemically similar but crystallographically different molecules in the asymmetric unit. Structural features of **2** are similar to those observed for **1**.

The FT-IR spectra of Compounds **1**–**4** showed weak absorptions in the range 3010–3177 cm^−1^, due to the azolyl ring C-H stretching and the presence of the BH_3_ moiety in Compounds **1** and **2** was detected by intense absorptions at 2255–2374 cm^−1^.

The ^1^H- and ^13^C-NMR spectra of **1** and **2** were recorded in CDCl_3_ and CD_3_OD, while the spectra of **3** and **4** were recorded in DMSO solution. Compounds **1**–**4** showed a single set of resonances for the imidazolium rings. The ^1^H NMR spectra of Compounds **1** and **2** at the 2-CH position does not show any reduced intensity after two days in CD_3_OD solution at room temperature, suggesting the absence of fast H-D exchange and therefore lack of deuteration at this position.

The ^11^B-NMR spectra showed a quartet at δ −19.38 and −19.21 ppm for Compounds **1** and **2**, respectively, in CDCl_3_ solution, indicating a coordination of the imidazole rings at the BH_3_ group [62,90]. The single broad ^11^B resonances observed at δ −6.52 ppm for Compound **3** and at δ −6.37 ppm for Compound **4**, in (CD_3_)_2_CO and CDCl_3_ solutions, respectively, are indicative of a four-coordinate boron center; they are in the range observed for analogously triphenylborate species [91], being considerably shifted in comparison with the triphenylborane one, which is observed at δ −60.2 ppm [92].

In the ESI(+)-MS spectra of **1** and **2** we observed peaks at *m*/*z* 195 and 223, due to the molecular specie [(HIm^Bn^)BH_3_ + Na]^+^ and [(HIm^Mes^)BH_3_ + Na]^+^, respectively. In addition the ESI(+)MS spectra displayed peaks due to the fragmentation species [HIm^R^ + H]^+^ and to the aggregates [(HIm^R^)_2_BH_2_]^+^ (R = Bn or Mes). Analogously, the ESI(+)-MS spectra of Compounds **3** and **4** were dominated by the peaks at *m/z* 83 and 159 due to the [HIm^CH3^ + H]^+^ and [HIm^Bn^ + H]^+^, respectively, along with a fragment at *m/z* 247 ([(HIm^CH3^)BPh_2_]^+^, 25%) and an aggregate at 481 ([(Im^Bn^)_2_BPh_2_]^+^, 45%), in **3** and **4** respectively.

**Scheme 5:**
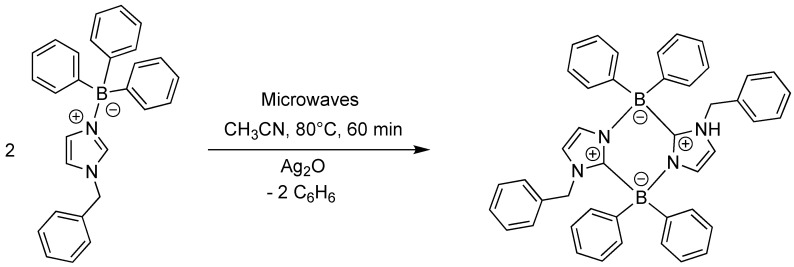
Microwave-assisted synthesis of Compound **5**.

Our aim was to synthesize new *N*-(alkyl/aryl)imidazolium-borates and study their reactivity and investigate their reactivity as carbene precursors in an effort to obtain borate-NHCs silver(I) complexes. However, treatment of Compounds **1**–**4** with ^n^BuLi to yield the imidazol-2-ylidenes always led to decomposition species. Further direct reactions of **1**–**4** with Ag_2_O, in different reaction conditions (r.t. or reflux; reaction times = 5, 24, 48, and 120 h; solvent = THF, CH_2_Cl_2_, CH_3_OH, and CH_3_CN), or with silver acetate (in CH_3_OH or CH_3_CN) to give silver carbene complexes were unsuccessful: only mixtures of unreacted or decomposition products were detected. The only partially isolable product of the reaction of **4** and Ag_2_O in CH_3_CN was the imidazabole species **5**. After these efforts, we found that the direct synthesis of imidazaboles [60,61,66] could be achieved by using microwave-assisted procedure [93], following a pre-set heating ramp of 1 h up to 80 °C, in technical-grade CH_3_CN and in the presence of Ag_2_O (Scheme 5). Unfortunately, this methodology was only successful for Compound **5**, and mixtures of products were obtained by using microwave-assisted procedure employing Compounds **1**–**3** as starting materials.

Compound **5** is an oil soluble in CH_3_OH, CH_3_CN and DMSO. Its formation can be explained by the abstraction of proton at 2-position of the imidazolium-triphenylborate and the successive bimolecular condensation of the produced anions with elimination of two benzene molecules (Scheme 5) [65]. Compound **5** has a framework of 1,4-diazonia-2,5-diboratacyclohexa-3,6-diene, which can also be regarded as an intramolecular carbene-borate adduct [59,60].

NMR spectra showed significant changes going from Compound **4** to the corresponding imidazabole species **5**. In particular, the ^1^H-NMR spectrum of **5** recorded in deuterated DMSO showed the disappearance of the diagnostic 2-C*H* imidazolium signal of **4** at 8.37 ppm upon cyclization. Analogously, in the ^13^C-NMR spectrum, the 2-*C*H imidazolium signal of **4** at 136.33 ppm was no longer observed in the spectrum of **5** that instead showed a new, albeit poorly intense, 2-C signal at 159.18 ppm indicative of the carbene-borate formation [94]. The remaining ^13^C-NMR data are very similar to those of **4**. The ^11^B-NMR spectrum contains a singlet at δ 1.43. The decreased ^11^B-NMR nuclear shielding in **5** as compared to **4** (δ^11^B −6.37) points towards lower delocalization of the positive charge in the imidazabole system [95].

**Scheme 6:**
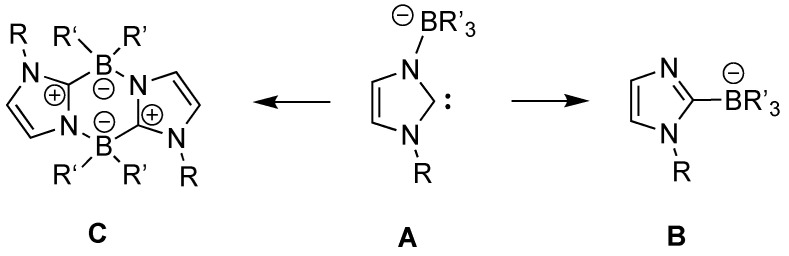
Rearrangement species (**B**,**C**) by isomerization or dimerization of the NHC-borate form (**A**).

Isomerization to the 2-borate imidazole forms by 1,2-BR_3_ migration [65], intramolecular addition/elimination or dimerization reactions may occur on deprotonation [57,58,59,60,61,66], presumably involving intermediates such as in Scheme 6A,B.

DFT studies by Vagedes et al. [57] suggested that direct interconversion of such anions by 1,2-migration is very unlikely. The borate substituent thermodynamically prefers to be bound to C-2 of the anionic heterocyclic moiety. Presumably, the Lewis acidic borane compensates the negative charge much more efficiently when bound to the carbon atom than when bound to the nitrogen atom, but their interconversion was precluded by a very high barrier of the respective 1,2-BR_3_ shift [57]. In particular, for Compound **5**, the probably initially generated “anionic Arduengo carbene” product **A** is proved unstable under the reaction conditions and it must be assumed that the rearrangement, experimentally observed to yield species C, is likely to have proceeded intermolecularly by two successive nucleophilic substitutions or by radical pathway as recently proposed by Chiu et al. [65].

As demonstrated in the BR_3_-functionalized NHC, the incorporation of anionic borate functionality enhances the donating ability of NHC [96,97]. However, we must conclude that the *N*-borato carbene anion A could exhibit its characteristic NHC chemistry when prepared or generated under conditions precluding intermolecular rearrangement pathways to their thermodynamically favored C(2)-borated imidazole isomers or head-to-tail imidazabole dimers.

## 3. Experimental Section

### 3.1. Materials and General Methods

All syntheses and handling were carried out under an atmosphere of dry oxygen-free dinitrogen, using standard Schlenk techniques or a glove box. Glassware was dried with a heat-gun under high vacuum. Solvents were purchased from commercial sources and purified by conventional methods prior to use. Elemental analyses (C, H, N, and S) were performed with a Fisons Instruments EA-1108 CHNS-O Elemental Analyzer (Thermo Fisher Scientific Inc., Waltham, MA, USA). Melting points were taken on an SMP3 Stuart Scientific Instrument (Bibby Sterilin Ltd., London, UK). IR spectra were recorded from 4000 to 400 cm^−1^ on a PerkinElmer Frontier FT-IR instrument (Perkin Elmer Inc., Waltham, MA, USA), equipped with single reflection universal diamond ATR top-plate. IR annotations used were as follows: br = broad, m = medium, mbr = medium broad, s = strong, sbr = strong broad, sh = shoulder, vs = very strong, w = weak, wbr = weak broad. ^1^H-, ^13^C-, and ^11^B-NMR spectra were recorded with an Oxford AS400 Varian spectrometer (400.4 MHz for ^1^H, 100.1 MHz for ^13^C, and 128.4 MHz for ^11^B) (Oxford Instruments, MA, USA) or with a 500 Bruker Ascend (500.1 MHz for ^1^H, 125 MHz for ^13^C, and 160.5 MHz for ^11^B) (Bruker BioSpin Corporation, 15 Fortune Drive, Billerica, MA, USA). Referencing was relative to tetramethylsilane (TMS) (^1^H and ^13^C) and BF_3_.Et_2_O (^11^B). NMR annotations used were as follows: br = broad; d = doublet, m = multiplet, s = singlet. Syntheses under microwave irradiation were performed by means of a Flexible Microwave Platform FlexSynth Milestone apparatus (Milestone Srl, Via Fatebenefratelli, Sorisole (BG), Italy). The reactions were performed in a 100-mL PTFE vessel, sealed using a Teflon crimp top. Electrospray mass spectra (ESI-MS) were obtained in positive—(ESI(+)MS) or negative-ion (ESI(−)MS) mode on an Agilent Technologies Series 1100 LC/MSD Mass Spectrometer (Agilent Technologies Inc, Santa Clara, CA, USA), using a methanol or acetonitrile mobile phase. The compounds were added to reagent grade methanol to give approximately 0.1 mM solutions, injected (1 µL) into the spectrometer via a Hewlett Packard 1090 Series II UV-Visible HPLC system (Agilent Technologies Inc, Santa Clara, CA, USA) fitted with an autosampler. The pump delivered the solutions to the mass spectrometer source at a flow rate of 300 mL min^−1^, and nitrogen was employed both as a drying and nebulizing gas. Capillary voltages were typically 4000 and 3500 V for the ESI(+)MS and ESI(−)MS modes, respectively. Confirmation of all major species in this ESI-MS study was supported by comparison of the observed and predicted isotope distribution patterns, the latter calculated using the IsoPro 3.1 computer program (T-Tech Inc., Norcross, GA, USA). 1-Benzylimidazole, 1-methylimidazole, BH_3_•THF complex, ammonium tetraphenylborate, and silver oxide were purchased from Sigma-Aldrich (Merck Life Science S.r.l., Via Monte Rosa, Milano, Italy). The 1-mesitylimidazole was synthesized in accordance with the literature method [98].

Caution! The materials used and synthesized in this study are energetic. They should be handled in quantities not exceeding the millimolar scale. Manipulations should be carried out behind blast shields and with adequate personal safety gear.

#### 3.1.1. Synthesis of (HIm^Bn^)BH_3_ (**1**)

1-Benzylimidazole (1.840 g, 11.631 mmol) was dissolved in dry THF (50 mL) under N_2_ atmosphere and BH_3_•THF complex (12.0 mL, 1M) was added drop by drop. The reaction mixture was stirred at room temperature for 24 h. Then, the volatiles were removed under reduced pressure to give a colorless oil. It was re-crystallized by CHCl_3_/diethyl ether/*n*-hexane (1/3/3) solution to obtain a white precipitate; it was filtered, washed with diethyl ether, and dried under reduced pressure to give **1** in 80% yield (1.601 g). Single crystals of **1** suitable for X-ray analysis were obtained by slow evaporation of a CHCl_3_ solution of **1**. Melting point: 92–94 °C. IR (cm^−1^): 3159w, 3135m, 3061w, 3038w (C-H); 2352m, 2297m, 2255m (B-H); 1540m, 1533m (C=C/C=N). ^1^H-NMR (CDCl_3_, 293 K): δ 2.2 (br, 3H, BH_3_), 5.13 (s, 2H, CH_2_Ph), 6.91 (s, 1H, 4-CH or 5-CH), 7.14 (s, 1H, 4-CH or 5-CH), 7.23–7.44 (m, 5H, C_6_H_5_), 7.79 (s, 1H, 2-CH). ^1^H-NMR (CD_3_OD, 293 K): δ 2.2 (qbr, 3H, BH_3_), 5.24 (s, 2H, CH_2_Ph), 7,03 (s, 1H, 4-CH or 5-CH), 7.19 (s, 1H, 4-CH or 5-CH), 7.26–7.43 (m, 5H, C_6_H_5_), 8.13 (s, 1H, 2-CH). ^13^C{^1^H}-NMR (CDCl_3_, 293 K): δ 52.35 (CH_2_Ph), 119.94, 127.98, 128.21, 129.33, 129.47, 133.46 (*C*H), 136.33 (2-*C*H). ^11^B{^1^H}-NMR (CDCl_3_, 293 K): δ −19.38 (s, BH_3_). ^11^B-NMR (CDCl_3_, 293 K): δ −19.38 (q, BH_3,_ J_B-H_ = 96 Hz). ESI-MS (major positive-ions, CH_3_OH), *m*/*z* (%): 159 (40) [HIm^Bn^ + H]^+^, 181 (40) [HIm^Bn^ + Na]^+^, 195 (90) [(HIm^Bn^)BH_3_ + Na]^+^, 329 (100) [(HIm^Bn^)_2_BH_2_]^+^. Anal. Calcd. for C_10_H_13_BN_2_: C 69.82, H 7.62, N 16.28%. Found: C 69.52, H 7.30, N 15.91%.

#### 3.1.2. Synthesis of (HIm^Mes^)BH_3_ (**2**)

1-mesityl-imidazole (0.930 g, 5.000 mmol) was dissolved in dry THF (30 mL) under N_2_ atmosphere and BH_3_•THF complex (5.2 mL, 1M) was added drop by drop. The reaction mixture was stirred at room temperature for 24 h. Then, the volatiles were removed under reduced pressure to give a brown oil. It was re-crystallized by CHCl_3_/diethyl ether/*n*-hexane (1/3/3) solution to obtain a brown precipitate; it was filtered, washed with diethyl ether, and dried under reduced pressure to give **1** in 68% yield (0.680 g). Single crystals of **2** suitable for X-ray analysis were obtained by slow evaporation of a CHCl_3_/THF solution of **2**. Melting point: 109–111 °C. IR (cm^−1^): 3177w, 3155w, 3132w, 3061w, 3028w (C-H); 2374m, 2338m, 2323m, 2300m, 2259m (B-H); 1526s (C=C/C=N). ^1^H-NMR (CDCl_3_, 293 K): δ 2.03 (s, 6H, CH_3_^Mes^), 2.3 (br, 3H, BH_3_), 2.37 (s, 3H, CH_3_^Mes^), 6.90 (s, 1H, 4-CH or 5-CH), 7.02 (s, 2H, CH^Mes^), 7.31 (s, 1H, 4-CH or 5-CH), 7.75 (s, 1H, 2-CH). ^1^H-NMR (CD_3_OD, 293 K): δ 2.04 (s, 6H, CH_3_^Mes^), 2.1 (br, 3H, BH_3_), 2.35 (s, 3H, CH_3_^Mes^), 7.08 (s, 2H, CH^Mes^), 7.23 (s, 1H, 4-CH or 5-CH), 7.25 (s, 1H, 4-CH or 5-CH), 8.14 (s, 1H, 2-CH). ^13^C{^1^H}-NMR (CDCl_3_, 293 K): δ 17.33, 21.06 (CH_3_^Mes^), 121.25, 128.12, 129.49, 131.71, 134.86, 136.85 (CH), 140.34 (2-CH). ^11^B-NMR (CDCl_3_, 293 K): δ −19.21 (dbr, BH_3_). ESI-MS (major positive-ions, CH_3_OH), *m/z* (%): 187 (15) [HIm^Mes^ + H]^+^, 223 (55) [(HIm^Mes^)BH_3_ + Na]^+^, 385 (100) [(HIm^Mes^)_2_BH_2_]^+^. Anal. Calcd. for C_12_H_17_BN_2_: C 72.03, H 8.56, N 14.00%. Found: C 71.81, H 8.25, N 13.60%.

#### 3.1.3. Synthesis of (HIm^CH3^)BPh_3_ (**3**)

A large excess of 1-methylimidazole (0.603 g, 7.344 mmol) was dissolved in acetonitrile (CH_3_CN, 60 mL). Then, ammonium tetraphenylborate (NH_4_BPh_4_, 1.770 g, 5.248 mmol) was added to the solution. A white precipitate was formed, but the solution became limpid after 1 h. The reaction proceeded for 70 h at reflux under magnetic stirring. At the end, the solution was dried at reduced pressure, obtaining a white solid. Et_2_O was added to the round-bottom flask to purify the residue from the starting materials that did not react. The resulting suspension was filtered, dried under reduced pressure, and furthe purified with CHCl_3_ to precipitate the excess of NH_4_BPh_4_. The mixture was filtered and the mother liquors were dried at reduced pressure to give the white ligand (HIm^CH3^)BPh_3_ (**3**) in 76% yield (1.293 g). Melting point: 209–212 °C. IR (cm^−1^): 3158w, 3133m, 3085w, 3064m, 3054mbr, 3010mbr (C-H); 1546m, 1531m, 1483mbr (C=C/C=N). ^1^H-NMR (DMSO-*d*_6_, 293 K): δ 3.79 (s, 3H, NCH_3_), 6.90 (d, 1H, 4-CH or 5-CH), 7.03–7.15 (m, 15H, CH), 7.44 (d, 1H, 4-CH or 5-CH), 8.09 (s, 1H, 2-CH). ^13^C{^1^H}-NMR (DMSO-d_6_, 293 K): δ 35.13 (NCH_3_), 122.34, 124.85, 126.42, 127.02, 134.49, 138.58 (CH). ^11^B-NMR (Acetone-*d*_6_, 293 K): δ −6.52 (s, BPh_3_). ESI-MS (major positive ions, CH_3_CN), *m*/*z* (%): 83 (100) [HIm^CH3^ + H]^+^, 247 (25) [(HIm^CH3^)BPh_2_]^+^. Anal. Calcd. for C_22_H_21_BN_2_: C 81.50, H 6.53, N 8.64. Found: C 81.14, H 6.56, N 8.38.

#### 3.1.4. Synthesis of (HIm^Bn^)BPh_3_ (**4**)

A large excess of 1-benzylimidazole (0.633 g, 4.000 mmol) was dissolved in CH_3_CN (60 mL). Then, NH_4_BPh_4_ (0.961 g, 2.850 mmol) was added to the solution. A white precipitate was formed, but the solution became limpid after 1 h. The reaction proceeded for 70 h at reflux under magnetic stirring. At the end, the solution was dried at reduced pressure, obtaining a white solid. EtOH was added to the round-bottom flask to purify the residue from the starting materials that did not react. The resulting suspension was filtered and dried at reduced pressure to give the white ligand (HIm^Bn^)BPh_3_ (**4**) in 50% yield (0.570 g). Melting point: 175–178 °C. IR (cm^−1^): 3163m, 3140m, 3125m, 3064mbr, 3023m (C-H); 1531mbr, 1506m, 1489mbr (C=C/C=N). ^1^H-NMR (DMSO-*d*_6_, 293 K): δ 5.39 (s, 2H, CH_2_Ph), 6.91 (s, 1H, 4-CH or 5-CH), 7.04–7.43 (m, 20H, C_6_H_5_), 7.49 (s, 1H, 4-CH or 5-CH), 8.37 (s, 1H, 2-CH). ^13^C{^1^H}-NMR (DMSO-*d*_6_, 293 K): δ 51.24 (CH_2_Ph), 121.25, 124.90, 127.23, 128.21, 129.33, 129.47, 133.46 (CH), 136.33 (2-CH). ^11^B-NMR (CDCl_3_, 293 K): δ −6.37 (s, BPh_3_). ESI-MS (major positive ions, CH_3_CN), *m*/*z* (%): 91 (80) [C_7_H_7_]^+^, 159 (100) [HIm^Bn^ + H]^+^, 242 (50) [BPh_3_ + H]^+^, 481 (45) [(Im^Bn^)_2_BPh_2_]^+^. Anal. Calcd. for C_28_H_25_BN_2_: C 84.01, H 6.29, N 7.00. Found: C 83.72, H 6.03, N 7.06.

#### 3.1.5. Synthesis of (Im^Bn^BPh_2_)_2_ (5)

In a 100-mL PTFE vessel equipped with a magnetic stir bar, Compound **4** (0.360 g, 0.900 mmol), silver oxide (Ag_2_O, 0.104 g, 0.450 mmol), and CH_3_CN (25 mL) were added. The reaction mixture was heated in the microwave reactor following a pre-set heating ramp, up to 80 °C. Once the temperature was reached, the reaction proceeded for 1 h and then it was cooled following a pre-set cooling ramp, to room temperature. All the steps were performed always under magnetic stirring. At the end, the mixture was filtered and the obtained mother liquors were dried at reduced pressure to give the oily brownish residue (Im^Bn^BPh_2_)_2_ (**5**) in 54% yield (0.157 g). IR (cm^−1^): 3161m, 3143m, 3113sh, 3087m, 3064m, 3038m, 3024m, 3010m, 2999m, 2972m, 2938wbr (C-H); 1600m, 1587m, 1571m, 1534s, 1509s, 1496sbr (C=C/C=N). ^1^H-NMR (DMSO-*d_6_*, 293 K): δ 5.18 (s, 2H, CH_2_), 6.91 (s, 1H, 4-CH or 5-CH), 7.18–7.36 (m, 15H, ArH), 7.77 (s, 1H, 4-CH or 5-CH). ^1^H-NMR (CDCl_3_, 293 K): δ 5.13 (s, 2H, CH_2_), 6.92 (s, 1H, 4-CH or 5-CH), 7.11–7.44 (m, 15H, ArH), 7.67 (s, 1H, 4-CH or 5-CH). ^13^C{^1^H}-NMR (DMSO-*d_6_*, 293 K): δ 50.04 (*C*H_2_Ph), 120.14, 127.98, 128.28, 128.79, 128.94, 129.17, 130.56, 134.47 (*C*H), 159.18 (2-C). ^11^B-NMR (DMSO-*d_6_*, 293 K): δ 1.43 (s). ESI-MS (major positive ions, CH_3_CN), *m*/*z* (%): 91 (95) [C_7_H_7_]^+^, 159 (100) [HIm^Bn^ + H]^+^. Elemental analysis for C_29_H_27_AgBN_2_ (%): calculated: H 5.94, C 82.01, N 8.69; found: H 6.04, C 81.27, N 8.89.

### 3.2. Crystallographic Data Collection and Refinement

A suitable crystal covered with a layer of hydrocarbon/Paratone-*N* oil was selected and mounted on a Cryo-loop and immediately placed in the low temperature nitrogen stream. X-ray intensity data were measured at 100(2) K on a Bruker SMART APEX II CCD area detector system equipped with an Oxford Cryosystems 700 series cooler, a graphite monochromator, and a Mo Kα fine-focus sealed tube (λ = 0.71073 Å). Intensity data were processed using the Bruker ApexII program suite. Absorption corrections were applied by using SADABS. Initial atomic positions were located by direct methods using XS, and the structures of the compounds were refined by the least-squares method using SHELXL [99]. All the non-hydrogen atoms were refined anisotropically. The hydrogen atoms attached to boron (B-H) were located in difference Fourier maps, included and refined freely with isotropic displacement parameters. All the other hydrogen atoms were placed at calculated positions and refined using a riding model. X-ray structural figures were generated using Olex2 [100]. The CCDC 2010217–2010218 contain the supplementary crystallographic data. These data can be obtained free of charge via http://www.ccdc.cam.ac.uk/conts/retrieving.html or from the Cambridge Crystallographic Data Centre (CCDC), 12 Union Road, Cambridge, CB2 1EZ, UK).

## 4. Conclusions

Two imidazolium-trihydridoborate adducts were obtained by addition of BH_3_•THF to *N*-benzyl- and *N*-mesitylimidazoles. In addition, two imidazolium-triphenylborates were obtained by displacement of one phenyl group of ammonium tetraphenylborate reacting with methyl- or benzyl-imidazoles. 3-Benzyl-imidazolium-1-yl)trihydridoborate and (3-mesityl-imidazolium-1-yl)trihydridoborate were also characterized by X-ray crystallography. The reactivity of these new compounds as carbene precursors was investigated and a new dimeric carbene-borate adduct was obtained via a microwave-assisted procedure. The intermolecular rearrangement pathway to the head-to-tail imidazabole dimer prevented the isolation of this type of compounds and the development of their characteristic NHC chemistry.
